# Supplementary material for: Content-rich biological network constructed by mining PubMed abstracts
Source: BMC Bioinformatics. 2004 Oct 8;5:147. doi: 10.1186/1471-2105-5-147 (PMC528731; doi:10.1186/1471-2105-5-147)
Supplement: Additional File 5 — The original Chilibot query results of the term "long-term potentiation (LTP)" and 22 other terms, limiting the latest references analyzed to the years 1990, 1995, 2000, and 2004. [file 1471-2105-5-147-S5.bz2 › chilibotAdditionalFile5/ltp2000/html/PI-3K_KV4.2.html]

 


 **PI-3K** and **KV4.2** 
  
Found 1 abstracts in PubMed,  **1 abstracts were retrieved and analyzed**.  


---

 Search Google  |
 PDF files only 
|  EDU domain only 

---

**Interactive relationship** (e.g. stimulation, inhibition, etc)

**Parallel relationship** (e.g. studied together, co-existance, homology, etc.)

- We observed that exposure to LY294002 or wortmannin, inhibitors of phosphatidylinositol PI 3 kinase  [ **PI-3K** ] , reduced somatic cross sectional area, neurite outgrowth, whole cell capacitance, IA amplitude and density amplitude normalized to membrane area, and immunoreactivity for  **Kv4.2**  and or Kv4.3 potassium channel subunits likely to be present in the channels carrying IA.  Ref: 9698319 J Neurosci, 1998
